# Extracellular nanovesicles released from the commensal yeast *Malassezia sympodialis* are enriched in allergens and interact with cells in human skin

**DOI:** 10.1038/s41598-018-27451-9

**Published:** 2018-06-15

**Authors:** Henrik J. Johansson, Helen Vallhov, Tina Holm, Ulf Gehrmann, Anna Andersson, Catharina Johansson, Hans Blom, Marta Carroni, Janne Lehtiö, Annika Scheynius

**Affiliations:** 1 Department of Oncology-Pathology, Karolinska Institutet, Science for Life Laboratory, 17121 Stockholm, Sweden; 2 Department of Clinical Science and Education, Södersjukhuset, Karolinska Institutet, and Unit Sachs’ Children and Youth Hospital, Södersjukhuset, SE-118 83, Stockholm, Sweden; 3 Department of Medicine Solna, Translational Immunology Unit, Karolinska Institutet and University Hospital, 17176 Stockholm, Sweden; 4 Advanced Light Microscopy Facility, Royal Institute of Technology, Science for Life Laboratory, 17121 Solna, Sweden; 5 Cryo-EM National Facility, Science for Life Laboratory, 17177 Stockholm, Sweden; 6 Clinical Genomics, Science for Life Laboratory, 17177 Stockholm, Sweden

**Keywords:** Clinical microbiology, Chronic inflammation, Fungal host response, Proteomics

## Abstract

*Malassezia sympodialis* is a dominant commensal fungi in the human skin mycobiome but is also associated with common skin disorders including atopic eczema (AE). *M*. *sympodialis* releases extracellular vesicles, designated MalaEx, which are carriers of small RNAs and allergens, and they can induce inflammatory cytokine responses. Here we explored how MalaEx are involved in host-microbe interactions by comparing protein content of MalaEx with that of the parental yeast cells, and by investigating interactions of MalaEx with cells in the skin. Cryo-electron tomography revealed a heterogeneous population of MalaEx. iTRAQ based quantitative proteomics identified in total 2439 proteins in all replicates of which 110 were enriched in MalaEx compared to the yeast cells. Among the MalaEx enriched proteins were two of the *M*. *sympodialis* allergens, Mala s 1 and s 7. Functional experiments indicated an active binding and internalization of MalaEx into human keratinocytes and monocytes, and MalaEx were found in close proximity of the nuclei using super-resolution fluorescence 3D-SIM imaging. Our results provides new insights into host-microbe interactions, supporting that MalaEx may have a role in the sensitization and maintenance of inflammation in AE by containing enriched amounts of allergens and with their ability to interact with skin cells.

## Introduction

Our skin is colonized by a diverse microbiota, including bacteria, viruses and fungi, and we are just at the beginning of understanding their role in influencing skin health. *Malassezia* is the most abundant fungal skin inhabitant of humans^1^, and colonizes the human skin right after birth^2^. The *Malassezia* species show lipid-dependency and lipolytic enzymes such as lipases are necessary for them to acquire fatty acids from the surroundings^1^. The *Malassezia* genus currently includes 17 species^1^. One of the most frequently isolated species from human skin is *M*. *sympodialis*, which besides being a commensal yeast is also associated with several common skin disorders such as atopic eczema (AE)^3^. AE is a complex inflammatory skin disorder, which is cyclical, with relapsing flare periods^4^, and affects 15 to 20% of young children and up to 3% of adults^5^. While the pathogenesis of the disease still remains unclear, studies suggest that a genetic predisposition in combination with environmental factors facilitate the development of AE^6^. A defective skin barrier in turn might assist the entry of microorganisms, such as *Malassezia* and their allergens. Thirteen *Malassezia* allergens have been sequenced so far^7^.

*M*. *sympodialis* may also affect AE via secreted extracellular vesicles (EVs), designated MalaEx^8^. EVs have been shown to be released both from different mammalian cell-types, and from microorganisms and parasites through the endosomal pathway or by budding from the plasma membrane^9–14^. By using vesicular transport, the content of the EVs are kept protected, and large molecules are enabled to cross the cell wall. Various types of EV ranging in size from 20 nm to 1,000 nm in diameter have been described and are classified mainly on their mechanisms of biogenesis and their physiological functions^10,15^. Those designated exosomes have been shown to be involved in immunoregulatory mechanisms such as immune activation and suppression, and intercellular communication^15^, whereas EV from microorganisms with thick cell walls, such as fungi, have been related to cytotoxicity, the invasion of host cells, and the transfer of virulence factors^11^. As seen with the heterogeneous secreted vesicles from the parasitic yeast stage of *Histoplasma capsulatum*, they can contain phospholipids and proteins associated to stress responses, pathogenesis, cell wall architecture and virulence^16^. Previously, we demonstrated that MalaEx are carriers of allergens^8^, like human dendritic or B cell-derived exosomes^17,18^, and can induce inflammatory cytokine responses with a significantly higher IL-4 production in peripheral blood mononuclear cells (PBMC) from patients with AE compared to healthy controls^8^, supporting the link between AE and *M*. *sympodialis*. Recently, we also identified the presence of small RNAs in MalaEx, which appeared to have an RNAi-independent route for biogenesis^19^.

We have previously performed mass spectrometry (MS) based proteomics on *M*. *sympodialis* cells for gene annotation^7,20^. In this study, we used large-scale quantitative proteomics characterization of *M*. *sympodialis* whole yeast cells (WC) and their released MalaEx to compare their content. Cryo-electron tomography was used to visualize MalaEx’s morphology, and possible interactions between MalaEx and cells in the skin were investigated to further explore MalaEx’s role in host-microbe interactions.

## Results

### Characterization of harvested MalaEx

MalaEx released from *M*. *sympodialis* cultured in RPMI medium for 48 h had a vesicle size of 171 ± 12 nm (mean ± SD; n = 5 different cultures) as measured with NanoSight. For proteomics we used MalaEx produced by *M*. *sympodialis* cultured for 72 h in mDixon broth to avoid contamination from fetal calf serum within the RPMI medium, which otherwise overrides the signals of less abundant proteins. These MalaEx had a mean size of 245 ± 10.9 nm (n = 4 different cultures) in line with Ryner *et al*.^19^.

**Figure 1:**
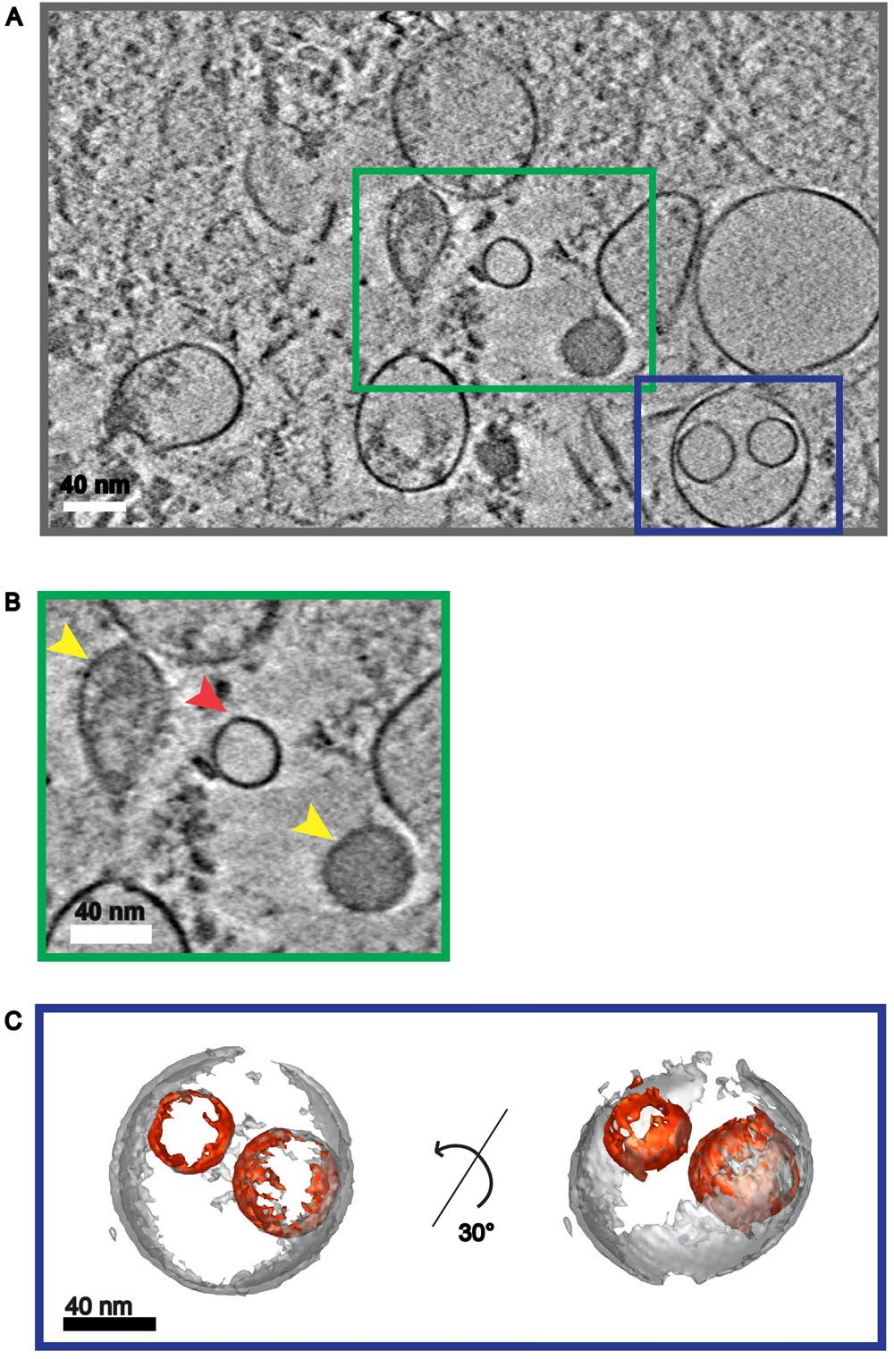
Cryo-electron tomography of MalaEx. **(A)** Central section of a tomographic reconstruction of MalaEx harvested from *M*. *sympodialis* cultured for 48 h in RPMI medium. The full tomogram is shown in Movie 1 (see Supplementary information). **(B)** Zoom into the green box of A showing a low electron density vesicle (red arrow) and high electron density vesicles with coated surfaces (yellow arrows). **(C)** Isosurface representation of the vesicles enclosed by the blue box in A. The surface representation shows two small vesicles with low electron density (in red) encircled by a larger one (in grey).

To further characterize the MalaEx we performed cryo-electron tomography. This technology revealed the presence of a heterogeneous population of vesicles with different sizes and internal vesicles, but also with varying electron-dense material suggesting different amount of internal content (Fig. 1A–C and Movie 1 Supplementary information). Some of the vesicles did also appear to have coatings, as seen on the surface of the filled vesicles in Fig. 1A,B.

### Proteomics analysis on MalaEx compared to parental cells

**Figure 2:**
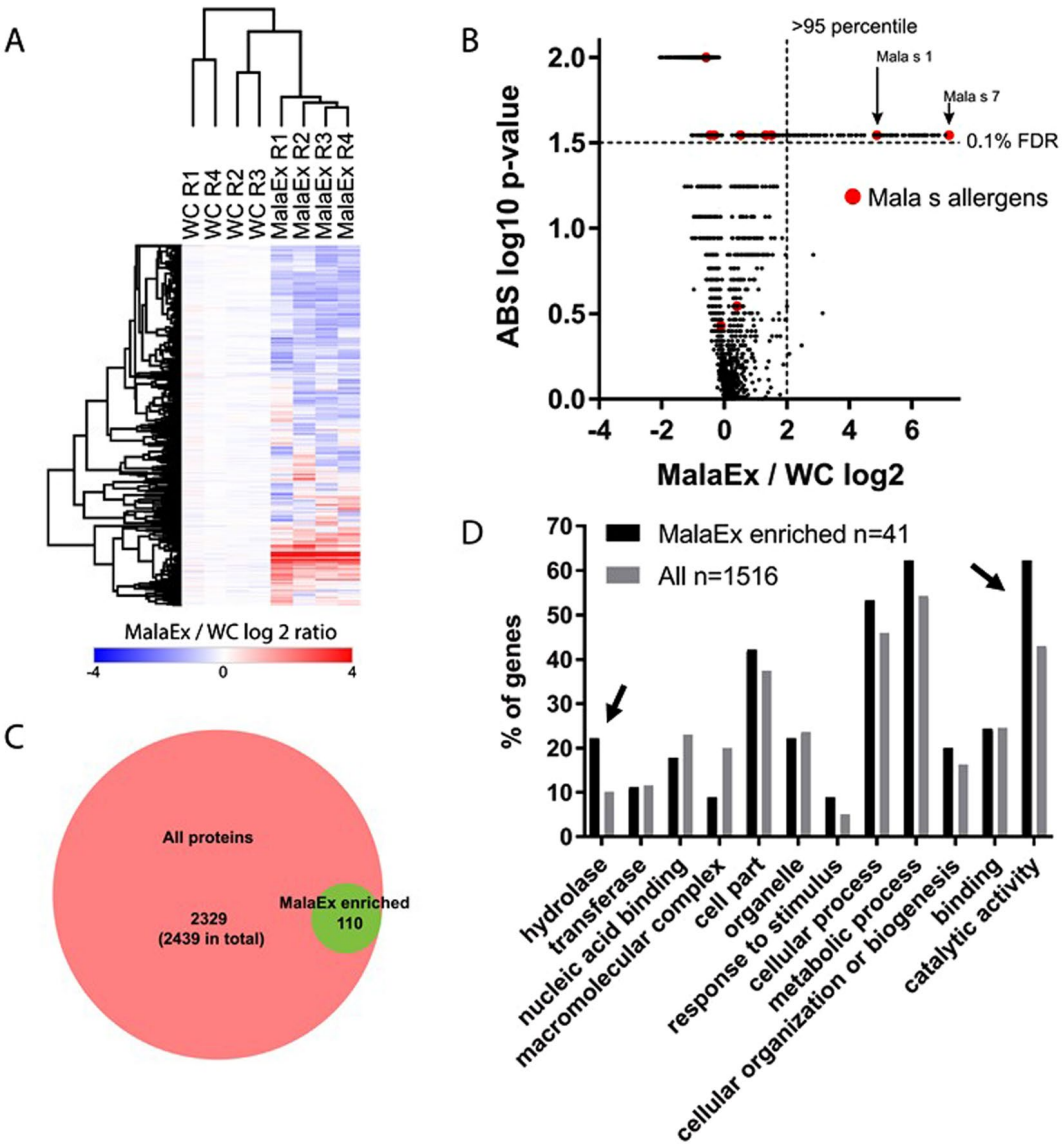
Characterizing MalaEx proteins by proteomics. (**A**) Quantitative overview of iTRAQ based proteomics experiments to define MalaEx enriched proteins by comparing MalaEx to *M*. *sympodialis* whole cells (WC). R 1–4 denotes the 4 biological replicates cultured for 72 h in mDixon broth, and 2439 proteins with quantification across all 8 samples are shown. (**B**) Volcano plot to define proteins enriched in MalaEx compared to WC. Proteomics data as in (**A**). Vertical and horizontal dashed lines indicate 95 percentile of ratios and q-value < 0.1, respectively. Mala s allergens are indicated in red. (**C**) Venn diagram depicting overlap between proteins considered enriched in MalaEx compared to WC. (**D**) Gene ontology (GO) analysis of MalaEx. GO term distribution in proteins defined as enriched in MalaEx (from **A**–**C**) compared to all identified proteins. Numbers indicate the number of *M*. *sympodialis* proteins that could be mapped to yeast homologs for the analysis. Arrows highlight proteins most enriched in MalaEx.

To define proteins that are enriched in MalaEx compared to WC we performed iTRAQ based quantitative proteomics comparing 4 replicates of WC cultured for 72 h, and MalaEx isolated from the their supernatants. We identified in total 3186 proteins from WC and MalaEx of which 2439 were quantified in all replicates (mainly due to missing values in the MalaEx quantification). A subset of proteins displayed higher levels in MalaEx compared to WC (Fig. 2A, Tables S1A and B). Using a combined fold change cutoff (>95 percentile) and p-value cutoff (t-test, permutation based, q-value <0.1), 110 proteins were considered enriched in MalaEx compared to WC (Fig. 2B,C, Tables S1A and B). Among these enriched proteins are 2 of the previously identified *M*. *sympodialis* allergens, Mala s 1 and Mala s 7 (Fig. 2B). In addition, the Mala s allergens 5, 6, 8–13 were identified but not considered enriched in MalaEx.

To obtain an understanding of the protein content of the MalaEx enriched proteins we used gene ontology (GO) terms based on *M*. *sympodialis* yeast homologs. The protein list of MalaEx enriched proteins (Table S1B) after conversion to yeast homologs was too short to obtain significant enrichment. However, displaying GO terms percentage wise for MalaEx and WC, there were more proteins with the GO terms hydrolase and catalytic activity in the enriched MalaEx proteins than in the whole list of proteins (Fig. 2D, Table 1).

**Table 1:** Gene Ontology (GO) terms and Panther classes for MalaEx enriched proteins and all identified proteins.

Category	Category name (Accession)	MalaEx enriched		All proteins in iTRAQ dataset	
		Genes (n)	Genes %^a^	Genes (n)	Genes %^a^
Panther class	hydrolase (PC00121)	10	22	177	10
	oxidoreductase (PC00176)	3	7	132	8
	enzyme modulator (PC00095)	2	4	104	6
	lyase (PC00144)	1	2	53	3
	transferase (PC00220)	5	11	203	12
	ligase (PC00142)	2	4	74	4
	nucleic acid binding (PC00171)	8	18	404	23
	transcription factor (PC00218)	1	2	57	3
	cytoskeletal protein (PC00085)	1	2	46	3
Cellular component	membrane (GO:0016020)	3	7	131	7
	macromolecular complex (GO:0032991)	4	9	352	20
	cell part (GO:0044464)	19	42	659	37
	organelle (GO:0043226)	10	22	415	24
Biological process	reproduction (GO:0000003)	1	2	6	0
	response to stimulus (GO:0050896)	4	9	89	5
	cellular process (GO:0009987)	24	53	809	46
	metabolic process (GO:0008152)	28	62	955	54
	biological regulation (GO:0065007)	3	7	85	5
	cellular component organization or biogenesis (GO:0071840)	9	20	286	16
Molecular function	binding (GO:0005488)	11	24	431	25
	structural molecule activity (GO:0005198)	2	4	174	10
	catalytic activity (GO:0003824)	28	62	756	43

^a^Percent of gene hit against total number of genes.

### Interactions of MalaEx with human keratinocytes and monocytes

**Figure 3:**
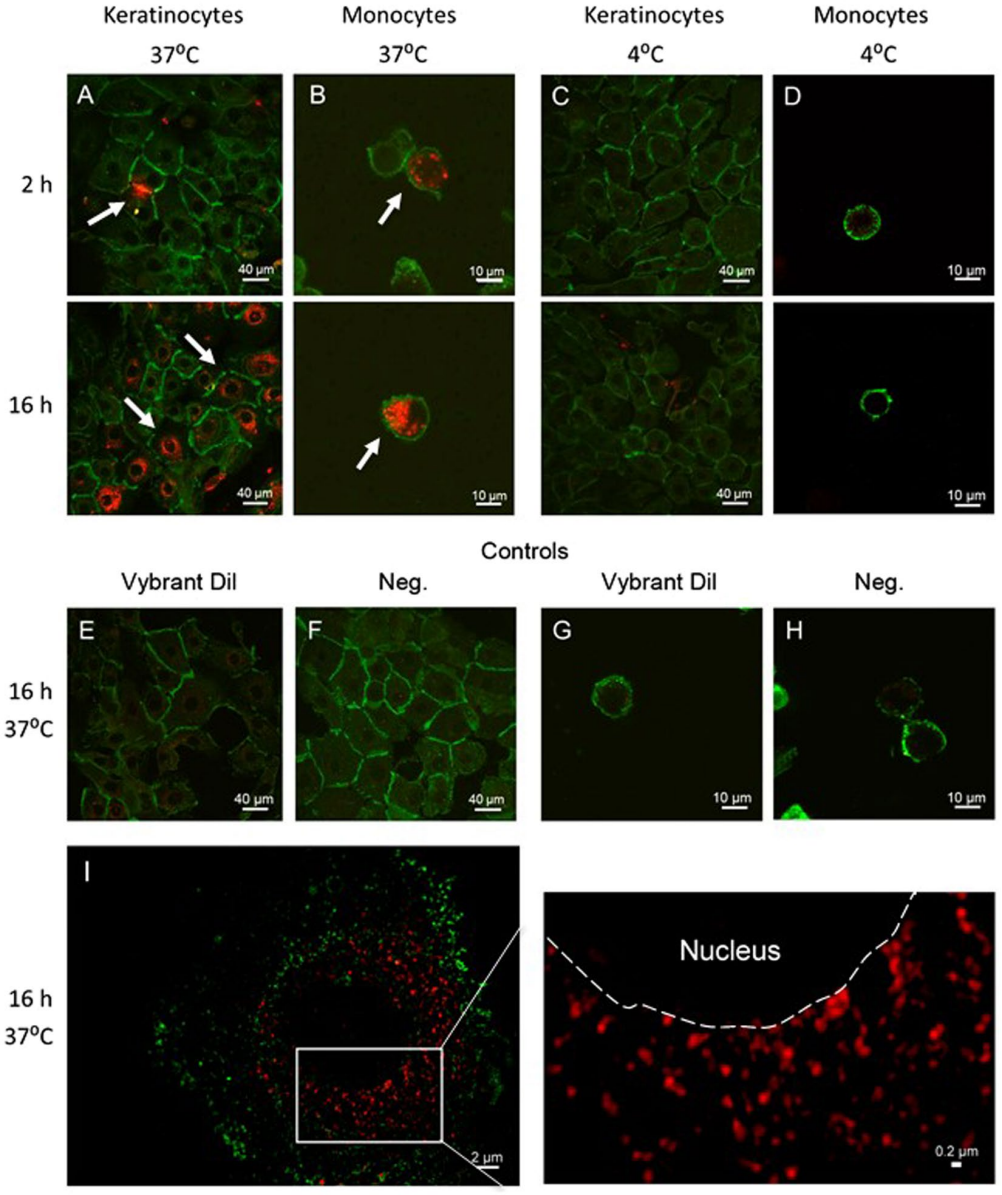
Cellular uptake of MalaEx in human primary keratinocytes and monocytes. (**A**–**H**) Confocal microscopy images following incubation of MalaEx, harvested from *M*. *sympodialis* cultured for 48 h in RPMI medium and Vybrant Dil labeled (red; 10 µg/ml), with (**A**,**C**) keratinocytes stained for E-cadherin (green) and (**B**,**D**) monocytes stained for CD14 (green) at 37 °C and 4 °C for 2 h and 16 h. Arrows highlight cells interacting with MalaEx. (**E**) Keratinocytes treated with Vybrant Dil control for 16 h at 37 °C and (**F**) cultured alone. (**G**) Monocytes treated with Vybrant Dil control for 16 h at 37 °C and (**H**) cultured alone. (**I**; left image) Structured Illumination Microscopy (3D-SIM) of keratinocytes incubated with MalaEx for 16 h at 37 °C from a single axial slice (depth 116 nm). (**I**; right image). Zoomed in 3D-SIM image of selected area (white square in left image) showing perinuclear localized MalaEx. Dashed line indicates the borderline between the nuclei and the cytoplasm of the keratinocyte.

To understand how MalaEx may be involved in host-microbe interactions, we studied MalaEx interactions with keratinocytes and monocytes. Keratinocytes are the main cell type of epidermis actively participating and orchestrating the innate immune response of the skin^21^ and monocytes are antigen-presenting cells, which are actively recruited at sites of chronic inflammation, such as damaged and inflamed skin as seen in AE^22^. MalaEx labeled with Vybrant Dil were incubated with keratinocytes or monocytes for 2 h or 16 h at 4 °C and 37 °C. Confocal laser scanning microscopy (CLSM) images demonstrated interactions between MalaEx and a few keratinocytes and an uptake of MalaEx by monocytes after 2 h at 37 °C (Fig. 3A and B). After 16 h both keratinocytes and monocytes had internalized increased amounts of MalaEx (Fig. 3A and B). Internalization of MalaEx was not observed at 4 °C, neither by keratinocyte (Fig. 3C), nor by monocytes (Fig. 3D) suggesting that an active mechanism is involved in the uptake seen at 37 °C. No background was seen when the control for potentially pelleted unbound dye was added to the cells for 16 h at 37 °C (Fig. 3E and G) or when the cells were cultured alone (Fig. 3F and H). Further analysis was performed with higher resolution using 3D-SIM on the 16 h co-cultures of MalaEx with keratinocytes at 37 °C, which demonstrated MalaEx mainly to be localized in the surrounding of the nuclei (Fig. 3I).

## Discussion

We have previously found that the commensal fungi *M*. *sympodialis* secretes extracellular vesicles (MalaEx), which are carriers of allergens^8^ and small RNAs^19^ and that they can induce inflammatory cytokine responses in human blood cells^8^. To further understand their role in host-microbe interactions, we present in this study new insights into the morphology of MalaEx, we performed the first proteome characterization of MalaEx and compared that with the whole *M*. *sympodialis* cells, and demonstrated interactions of MalaEx with skin cells.

In previous EM studies, which demonstrated a typical exosome-like morphology of MalaEx, sucrose gradient separation was used to isolate MalaEx at a density similar to that characterized for exosomes^8,19^. Here we decided to investigate the whole population of harvested vesicles prepared by serial ultracentrifugation and used cryo-electron tomography to be able to visualize 3D structures in more physiological conditions. Our results revealed a heterogeneous population of vesicles with different densities (Fig. 1). This is in agreement with reports on EVs from other fungal cells^16,23,24^, and from mammalian cell types such as platelets and mast cells^25,26^. The diversity of MalaEx suggests the existence of subpopulations of EVs’, which may have distinct mechanisms of biogenesis and release, and possess specific contents and thereby functions^11,23,24,27^. Further studies are needed to elucidate these aspects of different vesicles, which also require the development of relevant technologies^28^.

Quantitative proteomics identified in total 3186 proteins of which 2439 were quantified in all replicates and 110 proteins were enriched in MalaEx compared to the WC (Fig. 2). Proteomics of EV from other fungal species have identified between 100–400 proteins with large differences in protein identification between species^29^. A range of different GO terms were represented in both WC and MalaEx enriched proteins, as transferase, nucleic acid binding, cellular and metabolic processes, and cellular organization or biogenesis. Interestingly, our results indicate that proteins with the GO terms hydrolase and catalytic activity are enriched in MalaEx compared to WC (Fig. 2D). *M*. *sympodialis* is a lipid-dependent yeast, and therefore the ability to hydrolyze lipids from the environment by using lipolytic enzymes and integrate the fatty acids into the fungal cell are essential for their survival and are thought to play an important role in the pathogenesis of *Malassezia*^30,31^. By spreading hydrolases via MalaEx, such as lysophospholipases found in this study, in the surroundings of *M*. *sympodialis* may be beneficial for the survival of the yeast. Packaging of enzymes in EVs may e.g. bring the advantage of a higher digestive enzyme concentration for a more efficient acquisition of nutrients. However, possible consequences could be the degradation of the skin barrier. It is believed that lipase-mediated breakdown of sebaceous lipids and release of irritating unsaturated free fatty acids are important factors in the mechanism behind several skin disorders associated with *Malassezia*^32,33^.

It is still unknown how fungal vesicles traverse the cell wall. Here the EVs may also play an important role by carrying enzymes with the ability to hydrolyze cell wall components, which have the potential to promote cell wall reassembly for vesicle passage^34,35^. The yeast protein homologs with described functions as; aspartic proteinase yapsin, lysophospholipase, serine carboxypeptidase, acid phosphatase and glucan-glucosidase among the MalaEx proteins may be involved in degradation and passage of cell walls. Intriguingly, catalytic enzymes, such as DNA repair, ligase and helicases, histone deacetylase, ribonuclease and tRNA synthetase were identified among the MalaEx enriched proteins. They are essential for the survival of microorganisms, but the benefits of enriching them in MalaEx still need to be explored.

Furthermore, two of the *M*. *sympodialis* allergens, Mala s 1 and s 7, were found to be enriched in MalaEx (Fig. 2B). Both allergens encode proteins of unknown function without sequence homology to known allergens or to other known proteins and are predicted to be secreted^3,7^. Mala s 1 is mainly expressed on the cell surface of *Malassezia*^36^. A hypothesis, which merits further investigation, is that Mala s 1 via binding to phospoinositides is involved in membrane trafficking^37^ or may be involved in cell wall or post-secretory modifications of an as-yet-unidentified secondary metabolite, which may contribute to pathogenesis^7^. Interestingly, the characterized ortholog of Mala s 1 from the wheat pathogen *Fusarium graminearum* (Tri 14) has been proposed to be functionally associated with an adjacent gene cluster involved in biosynthesis of a mycotoxin^38^. Mala s 7 bears signal peptides and does not have transmembrane domains^7^. Genome sequencing has revealed a gene family amplification for the Mala s 7 allergen in *M*. *sympodialis*^7^. Notably, gene duplication is a major force driving evolution of new traits, including virulence. Thus, by spreading Mala s 1 and s 7, MalaEx may enhance host microbe interactions and may therefore have an in important role in the pathogenesis of AE.

EVs from mammalian cells have been found in diverse body fluids, such as blood^39^ and breast milk^40^, and represent important vehicles of intercellular communication in-between cells locally, but also at a distance^41^. Therefore, we hypothesize that MalaEx due to their small size compared to the whole yeast may be more efficient in disseminate biological signals to the host. Even though not shown yet, they might be able to reach through the outermost layer of skin, especially when the barrier is impaired such as in AE, and thereby being able to interact with host cells. Previously, it has been shown that monocyte-derived dendritic cells are able to engulf *M*. *sympodialis* and allergenic components from the yeast^42^. In this study, we were able to demonstrate for the first time that MalaEx interact with and are actively internalized by human primary monocytes and by keratinocytes. Since the uptake of MalaEx was not seen at 4 °C, it indicates that MalaEx were actively engulfed by endocytosis. Similar results have been reported for lysosome-derived EVs from melanocytes, which were endocytosed by keratinocytes^43^. Whether the role of the perinuclear localized MalaEx in keratinocytes is to deliver RNAs or proteins for intervening with the host cells, and if the interactions cause inflammation that contributes to skin diseases remains to be elucidated in future studies^17,30,38^. It has been suggested that vesicular secretion is a general mechanism in fungi for the transport of macromolecules related to virulence^16^. However, *Malassezia* may also have beneficial effects for the host. Recently, it was reported that *Malassezia globosa*, by its ability to secrete an aspartyl protease, MgSAP1, could inhibit *Staphylococcus aureus* biofilm formation, a virulence attribute^44^. Whether MalaEx participate in beneficial outcomes for the host is a topic for further studies.

## Conclusions

The results of the present study offer an increased understanding of the nature of MalaEx, which seem to be morphological diverse, are enriched with certain proteins, and have the ability to interact with skin cells. These results gives us further clues for understanding more about host-microbe interactions in the sensitization and maintenance of the inflammation in AE, which is important for identifying prospective prevention and new potential therapeutic targets.

## Methods

### Malassezia sympodialis culture conditions

*M sympodialis* (ATCC 42132) was cultured on Dixon agar plates^45^ modified to contain 1% (vol/vol) Tween 60, 1% (wt/vol) agar, and no oleic acid (mDixon) at 32 °C. After 4 days cells were harvested, and re-suspended in PBS and counted in a Bürker chamber using trypan blue exclusion. 2 × 10^6^ yeast cells/ml was cultured in RPMI 1640 supplemented with penicillin 100 units/ml, streptomycin 100 µg/ml, 2 mM L-glutamine, and 10% heat inactivated fetal calf serum (all from Gibco BRL, Life Technologies Ltd, Paisley, UK) incubated for 48 h in 6% CO_2_ at 37 °C, as previously described^8^.

For proteomic analysis 2 × 10^6^ yeast cells/ml was cultured in mDixon broth supplemented with 100 units/ml penicillin and 100 µg/ml streptomycin (Gibco BRL) to avoid extensive protein contaminations derived from fetal calf serum used in the RPMI medium, and cultured for 72 h at 32 °C at 200 rpm.

Prior usage, mDixon broth and fetal calf serum had been ultracentrifuged overnight at 100 000 × g followed by filtration through a 0.22 µm filter (Nordic Biolabs, Täby, Sweden) to remove possible EV contaminants. At each culture step blood and Sabourand agar plates were inoculated in parallel to exclude bacterial and *Candida* contaminations, respectively.

### Collection of *M*. *sympodialis* cells for proteomics

At the end of the 72 h culture period in mDixon broth, the numbers of *M*. *sympodialis* cells were counted using trypan blue exclusion. Thereafter, the culture was pelleted at 1.200 × g for 5 min, washed twice in PBS and frozen at −20 °C for future preparation of cell lysate. The culture supernatant was taken care of for further MalaEx preparations.

### MalaEx preparations

*M*. *sympodialis*-derived nanovesicles (MalaEx) were prepared by serial ultracentrifugation. The culture supernatants were spun at 1.200 × g for 5 min followed by 3.000 × g for 30 min, and thereafter for the RPMI cultures filtered through a 0.22 µm filter (Nordic Biolabs) and frozen to −80 °C until further preparation. After thawing at RT, supernatants were centrifuged at 10.000 × g for 30 min. Thereafter, MalaEx were pelleted from the supernatants at 100.000 × g for 90 min, re-suspended in PBS and pelleted again at 100.000 × g for 90 min. The resulting pellet was carefully re-suspended in 100 µl PBS. Protein content was measured using a detergent compatible (DC) protein assay according to the manufacturer’s instructions (BioRad, Hercules, CA, USA). The MalaEx preparations were stored frozen at −80 °C.

### NanoSight analysis

The particle size of the MalaEx preparations was measured using a LM10 platform with sCMOS camera from NanoSight Ltd, Amesbury, UK. The system is equipped with a 405 nm laser running NTA 2.3 analytical software package. The samples were analyzed at 500 × dilution in PBS with camera level 14 and detection threshold 6. Three consecutive videos were recorded for each sample using batch control to automate the procedure. The temperature was set at 20 °C before acquisition.

### Cryo-electron tomography

Vitrification of MalaEx isolated from the RPMI cultures was performed with a Vitrobot (FEI Company; OR, USA). The samples were blotted for 3 sec and plunge frozen in liquid ethane. Tilt series were collected with SerialEM^46^ at 3° increments over a range of ±60°, with a defocus of 4 to 6 µm. Images were recorded on a 200 kV Talos Arctica (FEI Company) using a Falcon 2 direct electron detector (FEI) at a nominal magnification of 22 000× (corresponding to a pixel size of 6.61 Å). Fiducial-free alignment of tilt images and tomogram reconstructions was carried out in IMOD using the patch-track procedure^47^. Four tomograms were reconstructed and non-linear anisotropic diffusion (NAD) filtered^48^. Two tomogram were collected with the Volta phase plate using the Tomo software (FEI company) and reconstructed with Inspect3D (FEI company). Movies were generated using Fiji-ImageJ (imagej.net/Fiji open source image processing package).

### Preparation of M. sympodialis and MalaEx lysates for proteomics

#### Cell lysate

*M*. *sympodialis* cells stored at −20 °C were thawed, dissolved in 40 ml PBS and pelleted at 3000 × g for 5 min. This was repeated once to wash the cells. The final pellets were dissolved in 200 µl PBS and added to appr. 200 µl 0.4–0.6 mm silica beads (washed with acidified water to remove fine particles, dust, and other impurities typically found in untreated beads) and homogenized in Precellyse 24 homogenizer (Bertin Technologies, Montigny-le-Bretonneux, France), 5 cycles (6000 rpm, 3 × 30 s). The beads were allowed to settle and 100 µl cell lysate from every sample was transferred to a new tube. 100 µl 2x lysis buffer was added (yielding final concentration of 4% SDS, 0.1 mM DTT, 25 mM Hepes pH 7.6) followed by heating for 5 min at 95 °C and sonication 3 × 20 s. Protein concentration was measured with the DC protein assay (BioRad). 100 µl of lysate from *M*. *sympodialis* cells were stored at −80 °C before proteomic analysis.

#### MalaEx lysate

A 1:1 volume of 2x lysis buffer (yielding final concentration of 4% SDS, 0.1 mM DTT, 25 mM Hepes pH 7.6) was added to the MalaEx preparations followed by heating for 5 min at 95 °C and sonication 3 × 20 s. Protein concentration was measured with the DC protein assay (BioRad) and the lysates were further analyzed with proteomic techniques.

### Sample preparation for mass spectrometry

Biological fourplicates of either WC or MalaEx with equal amounts of protein were mixed with 1 mM DTT, 8 M urea, 25 mM HEPES, pH 7.6 in a centrifugation filtering unit, 10 kDa cutoff (Nanosep® Centrifugal Devices with Omega™ Membrane, Pall, New York, USA), and centrifuged for 15 min, 14.000 g, followed by another addition of 8 M urea buffer and centrifugation. Proteins were alkylated by 50 mM IAA, in 8 M urea, 25 mM HEPES, pH 7.6 for 10 min, centrifuged, followed by 2 more additions and centrifugations with 4 M urea, 25 mM HEPES pH 7.6. Trypsin (Promega). 1:50, trypsin:protein, was added to the samples in 0.250 M urea, 25 mM HEPES and digested overnight at 37 °C. The filter units were centrifuged for 15 min, 14.000 g, followed by another centrifugation with MQ and the flow-through was collected. iTRAQ 8plex labeling of the peptides were done according to the manufacturer’s protocol (Applied Biosystems) and cleaned by a strata-X-C-cartridge (Phenomenex, Værløse, Denmark).

### IPG-IEF of peptides

The iTRAQ labelled peptides, were separated by immobilized pH gradient - isoelectric focusing (IPG-IEF) on a pH 3–10 strip as described by Branca *et al*.^49^. Peptides were extracted from the strips by a prototype liquid handling robot, supplied by GE Healthcare Bio-Sciences AB. A plastic device with 72 wells were put onto each strip and 50 µl of MQ was added to each well. After 30 min incubation, the liquid was transferred to a 96 well plate and the extraction was repeated 2 more times. The extracted peptides were dried in speed vac and dissolved in 3% acetronitrile (ACN), 0.1% formic acid.

### LC-ESI-LTQ-Orbitrap analysis

Before analysis on the LTQ Orbitrap Velos (Thermo Fisher Scientific, San Jose, CA, USA), peptides were separated using an Agilent 1200 nano-LC system. Samples were trapped on a Zorbax 300SB-C18, and separated on a NTCC-360/100-5-153 (Nikkyo Technos., Ltd, Tokyo, Japan) column using a gradient of A (5% DMSO, 0.1% FA) and B (90% ACN, 5% DMSO, 0.1% FA), ranging from 3% to 40% B in 50 min with a flow of 0.4 µl/min. The LTQ Orbitrap Velos was operated in a data dependent manner, selecting 5 precursors for sequential fragmentation by CID and HCD, and analyzed by the linear iontrap and orbitrap, respectively. The survey scan was performed in the Orbitrap at 30.000 resolution (profile mode) from 300–2000 m/z, with AGC set to 1 × 10^6^ ions. For generation of HCD fragmentation spectra, a max ion injection time of 500 ms and AGC of 5 × 10^4^ were used before fragmentation at 37.5% normalized collision energy. For FTMS MS2 spectra, normal mass range was used, centroiding the data at 7500 resolution. Peptides for CID were accumulated for a max ion injection time of 200 ms and AGC of 3 × 10^4^, fragmented with 35% collision energy, wideband activation on, activation q 0.25, activation time 10 ms before analysis at normal scan rate and mass range in the linear iontrap. Precursors were isolated with a width of 2 m/z and put on the exclusion list for 90 s. Single and unassigned charge states were rejected from precursor selection.

### Peptide and protein identification

Orbitrap raw MS/MS files were converted to mzML format using msConvert from the ProteoWizard tool suite^50^. Spectra were then searched using MSGF+ (v10072)^51^ and Percolator (v2.08)^52^, where search results from 8 subsequent fractions were grouped for Percolator target/decoy analysis. All searches were done against the combined database of *M*. *sympodialis*^20^ and Bos Taurus (Uniprot 170217) in the Galaxy platform^53^. MSGF+ settings included precursor mass tolerance of 10 ppm, fully-tryptic peptides, maximum peptide length of 50 amino acids and a maximum charge of 6. Fixed modifications were iTRAQ, on lysines and peptide N-termini, and carbamidomethylation on cysteine residues, a variable modification was used for oxidation on methionine residues. Quantification of iTRAQ reporter ions was done using OpenMS project’s IsobaricAnalyzer (v2.0)^54^. PSMs found at 1% FDR (false discovery rate) were used to infer gene identities.

Protein quantification by iTRAQ reporter ions was calculated using ratios to the whole cell pool and normalized to the sample median. The median PSM reporter ratio from peptides unique to a protein group was used for quantification. Protein false discovery rates were calculated using the picked-FDR method using gene symbols as protein groups and limited to 1% FDR^55^. Identifications from Bos taurus were considered contaminants from culturing and removed.

### GO enrichment analysis

*M*. *sympodialis* yeast homologs was used to map GO terms to *M*. *sympodialis* proteins^20^. Panther was used to assign GO terms for the distribution of GO terms in MalaEx enriched proteins^56^.

### Co-cultures of MalaEx with human primary keratinocytes and monocytes

Epidermal keratinocytes were isolated from human abdomen *ex vivo* skin received from local hospitals according to manufacturer’s instructions (Gibco Invitrogen Corporation, Paisley, UK). In short, thin skin tissues were prepared by using a dermatome, which thereafter were incubated in Dispase solution (25 caseinolytic units/ml; Gibco Invitrogen Corporation) for 18 h at 4 °C. Epidermis was separated from dermis, and placed into 0.05% Trypsin-EDTA (Gibco Invitrogen Corporation) for 15 min at 37 °C for cell dissociation. After addition of Soybean Trypsin Inhibitor (Gibco Invitrogen Corporation) at a concentration of 10 mg/ml, cells were pelleted, washed and suspended in complete serum-free Keratinocyte-SFM medium supplemented with 100 IU/ml penicillin, 100 µg/ml streptomycin and 60 µg/ml Amphotericin B (Gibco Invitrogen Corporation). Approximately 3 × 10^6^ cells where seeded in 15 ml culture medium in each T-75 culture flask and cultured at 37 °C, 6% CO_2_. When cell confluence reached approximately 75%, the cells were sub-cultured in new culture flasks or seeded directly onto chamber culture slides (Falcon, Corning Incorporated, NY, USA) and used for experiments.

Monocytes were achieved by first separating peripheral blood mononuclear cells (PBMC) from a buffy coat derived from one healthy blood donor (Karolinska University Hospital Blood Bank, Stockholm, Sweden) by standard gradient centrifugation with Ficoll Paque (Amersham Pharmacia Biotech AB, Uppsala, Sweden), and thereafter by separating monocytes from PBMC by using anti-CD14 microbeads (Miltenyi Biotech, Bergisch Gladbach, Germany) according to the manufacturer’s instructions. Isolated cells were analyzed by flow cytometry (FACS Canto II; BD Biosciences, CA, USA) to check for CD14^+^ cell purity (FITC anti-human CD14 Ab, BioLegend, San Diego, CA, USA), which was 99% and the viability was 98% as analyzed by trypan blue exclusion. Monocytes were cultured in RPMI 1640 medium (Gibco Invitrogen Corporation), supplemented with 2 mM L-glutamine (Gibco Invitrogen Corporation), 100 IU/ml penicillin, 100 µg/ml streptomycin, and 10% heat inactivated fetal calf serum (Gibco) from which exosomes had been deleted by centrifugation at 100 000 g for 16 h and filtration trough a 0.22 µm filter.

MalaEx harvested from the RPMI cultures were stained with the red fluorescent membrane dye Vybrant Dil cell-labelling solution (Life Technologies, OR, USA) according to the manufacturer’s instructions, and thereafter washed twice by centrifugation at 100,000 × g (NVT90 rotor, Beckman Coulter, Indianapolis, IN, USA). As a control the same concentration of Vybrant Dil cell-labelling solution was centrifuged in parallel to create a background control for potentially pelleted unbound dye. Ten µg MalaEx/ml were added to monocytes (0.75 cells/ml) in RPMI medium, and to human primary keratinocytes which had been pre-seeded at a concentration of 0.15 × 10^6^ cells/ml and had reached 75% confluence onto chamber culture slides (Falcon, Corning Incorporated) in serum-free Keratinocyte-SFM (Gibco Invitrogen Corporation). Co-cultures were performed for 2 and 16 h at 37 °C or 4 °C. The study was approved by the regional ethical review board in Stockholm (2015/2082-31/1), and written informed consent was obtained from all subjects donating skin. All experiments were performed in accordance with the Helsinki Declaration ethical principles for medical research.

### Confocal laser-scanning microscopy (CLSM)

Co-cultures on the chamber culture slides of MalaEx with keratinocytes were fixed in cold acetone for 5 min and with monocytes in formaldehyde for 15 min. Keratinocytes were stained by a mouse anti-36/E-Cadherin (BD Biosciences), followed by a secondary anti-mouse IgG antibody conjugated with FITC (BD Biosciences). Monocytes co-cultured with MalaEx were stained with anti-CD14 conjugated with Alexa fluor 488 (Bioss Antibodies Inc., MA, USA). Glass slides were mounted with Vectashield mounting medium (Vector Laboratories, CA, USA) and fluorescent images as z-scans were acquired on a CLSM (TCS SP2; Leica Microsystems, Mannheim, Germany).

### Structured illumination microscopy (3D-SIM)

Super-resolution fluorescence 3D-SIM imaging^57^ was performed on a Zeiss Elyra PS.1 (Carl Zeiss, Jena, Germany) of the glass slides with MalaEx and keratinocytes co-cultures prepared for CLSM. Images were captured with an Andor iXon DU 855 EMCCD camera (Andor, Belfast, UK) with a Plan-Apochromate 100×/1.46 NA oil immersion objective. Excitation laser wavelengths used were 488 and 561 nm and fluorescence emission was collected through appropriate dichroic mirrors and single color bandpass filters (E-Cadherin 495–550 nm and MalaEx 570–620 nm). An EMCCD-gain of 10 to 20 and 5 grid rotations were applied with camera integration times of 100 to 150 ms. Image stacks with 50 nm lateral pixel size and 116 nm axial step size were sequentially acquired. Calibration measurements on 40 nm green fluorescent beads delivering a maximal focal resolution of 100 nm laterally and 250 nm axially. Raw SIM datasets were processed with the integrated ELYRA PS.1 system’s analysis software (Zen 2012 SP5 Black) with selection of automatic settings for SIM evaluations (i.e. theoretical PSF, noise filter setting, frequency weighting, baseline handling, etc.). After processing, rendered SIM images were checked for possible artefacts (e.g., amplified honeycomb noise patterns) to confirm suitable automatic analysis settings^58^.

### Statistical analysis

Gene-E (https://software.broadinstitute.org/GENE-E/) was used for making heatmaps and defining enriched proteins in MalaEx vs. WC comparison, using permutation based t-test. Proteins were considered enriched if the ratio between MalaEx and WC was above the 95 percentile and below 0.1 false discovery rate (FDR). Graphpad Prism 7 was used to make figures.

### Data access

MS proteomics data is deposited to jPOSTrepo (a repository that is in the ProteomeXchange consortium) with the dataset identifier JPST000288 & PXD009113. MS data can be previewed before publication at: https://repository.jpostdb.org/preview/13203449215a9d422a6ffc5 Using the code: 2889

## Electronic supplementary material

Supplementary information

Movie 1 Tomogram of purified MalaEx vesicles.

Table S1A and B
