# Author Correction: Extracellular nanovesicles released from the commensal yeast *Malassezia sympodialis* are enriched in allergens and interact with cells in human skin

**DOI:** 10.1038/s41598-019-43724-3

**Published:** 2019-10-15

**Authors:** Henrik J. Johansson, Helen Vallhov, Tina Holm, Ulf Gehrmann, Anna Andersson, Catharina Johansson, Hans Blom, Marta Carroni, Janne Lehtiö, Annika Scheynius

**Affiliations:** 1 Department of Oncology-Pathology, Karolinska Institutet, Science for Life Laboratory, 17121 Stockholm, Sweden; 2 Department of Clinical Science and Education, Södersjukhuset, Karolinska Institutet, and Unit Sachs’ Children and Youth Hospital, Södersjukhuset, SE-118 83 Stockholm, Sweden; 3 Department of Medicine Solna, Translational Immunology Unit, Karolinska Institutet and University Hospital, 17176 Stockholm, Sweden; 4 Advanced Light Microscopy Facility, Royal Institute of Technology, Science for Life Laboratory, 17121 Solna, Sweden; 5 Cryo-EM National Facility, Science for Life Laboratory, 17177 Stockholm, Sweden; 6 Clinical Genomics, Science for Life Laboratory, 17177 Stockholm, Sweden

Correction to: *Scientific Reports* 10.1038/s41598-018-27451-9, published online 15 June 2018

This Article contains a typographical error regarding the concentration of Amphotericin B in the Methods section under subheading ‘Co-cultures of MalaEx with human primary keratinocytes and monocytes’ where,

“After addition of Soybean Trypsin Inhibitor (Gibco Invitrogen Corporation) at a concentration of 10 mg/ml, cells were pelleted, washed and suspended in complete serum-free Keratinocyte-SFM medium supplemented with 100 IU/ml penicillin, 100 µg/ml streptomycin and 60 µg/ml Amphotericin B (Gibco Invitrogen Corporation).”

should read:

“After addition of Soybean Trypsin Inhibitor (Gibco Invitrogen Corporation) at a concentration of 10 mg/ml, cells were pelleted, washed and suspended in complete serum-free Keratinocyte-SFM medium supplemented with 100 IU/ml penicillin, 100 µg/ml streptomycin and 0.5 µg/ml Amphotericin B (Gibco Invitrogen Corporation).”

