# Supplementary material for: Extracellular nanovesicles released from the commensal yeast Malassezia sympodialis are enriched in allergens and interact with cells in human skin
Source: Sci Rep. 2018 Jun 15;8:9182. doi: 10.1038/s41598-018-27451-9 (PMC6004016; doi:10.1038/s41598-018-27451-9)
Supplement: Supplementary file 1 — Supplementary information [file 41598_2018_27451_MOESM1_ESM.pdf]

Supplementary Information

Extracellular nanovesicles released from the commensal yeast *Malassezia sympodialis* are enriched in allergens and interact with cells in human skin

Henrik J. Johansson[1§], Helen Vallhov[2§], Tina Holm[3], Ulf Gehrmann[3], Anna Andersson[3], Catharina Johansson[2], Hans Blom[4], Marta Carroni[5], Janne Lehtiö[1], & Annika Scheynius[2,6]

[1]Department of Oncology-Pathology, Karolinska Institutet, Science for Life Laboratory, 17121 Stockholm, Sweden
[2]Department of Clinical Science and Education, Södersjukhuset, Karolinska Institutet, and Unit Sachs' Children and Youth Hospital, Södersjukhuset, SE-118 83 Stockholm, Sweden
[3]Department of Medicine Solna, Translational Immunology Unit, Karolinska Institutet and University Hospital, 17176 Stockholm, Sweden
[4]Advanced Light Microscopy Facility, Royal Institute of Technology, Science for Life Laboratory, 17121 Solna, Sweden
[5]Cryo-EM National Facility, Science for Life Laboratory, 17177 Stockholm, Sweden
[6]Clinical Genomics, Science for Life Laboratory, 17177 Stockholm, Sweden

[§]These authors contributed equally to this work
[*]Corresponding author:
Annika Scheynius
Department of Clinical Science and Education
Södersjukhuset, Karolinska Institutet
SE-118 83 Stockholm, Sweden
Phone: +46 (0)70 6057927
E-mail: annika.scheynius@ki.se

Movie 1: Tomogram of purified MalaEx vesicles. Full tomogram showing MalaEx, harvested from *M. sympodialis* cultured for 48 h, with low or high electron density, and with vesicles encircled by a larger one. Filament like structures and small protein debris derived from the preparation are also visible.

Table S1A. iTRAQ based quantitative proteomics to define MalaEx enriched proteins.

Table S1B. MalaEx enriched proteins.
